# Foundations of Emergency Medicine: Impact of a Standardized, Open-access, Core Content Curriculum on In-Training Exam Scores

**DOI:** 10.5811/westjem.18387

**Published:** 2024-03-25

**Authors:** Jaime Jordan, Natasha Wheaton, Nicholas D. Hartman, Dana Loke, Nathaniel Shekem, Anwar Osborne, P. Logan Weygandt, Kristen Grabow Moore

**Affiliations:** * University of California Los Angeles, David Geffen School of Medicine, Department of Emergency Medicine, Los Angeles, California; † Wake Forest University School of Medicine, Department of Emergency Medicine, Winston-Salem, North Carolina; ‡ Northwestern University Feinberg School of Medicine, Department of Emergency Medicine, Chicago, Illinois; § University of Iowa, Department of Emergency Medicine, Iowa City, Iowa; ∥ Emory University, Department of Emergency Medicine, Atlanta, Georgia; ¶ Johns Hopkins University School of Medicine, Baltimore, Maryland

## Abstract

**Introduction:**

Learners frequently benefit from modalities such as small-group, case-based teaching and interactive didactic experiences rather than passive learning methods. These contemporary techniques are features of Foundations of Emergency Medicine (FoEM) curricula, and particularly the Foundations I (F1) course, which targets first-year resident (PGY-1) learners. The American Board of Emergency Medicine administers the in-training exam (ITE) that provides an annual assessment of EM-specific medical knowledge. We sought to assess the effect of F1 implementation on ITE scores.

**Methods:**

We retrospectively analyzed data from interns at four EM residency programs accredited by the Accreditation Council for Graduate Medical Education. We collected data in 2021. Participating sites were geographically diverse and included three- and four-year training formats. We collected data from interns two years before (control group) and two years after (intervention group) implementation of F1 at each site. Year of F1 implementation ranged from 2015–2018 at participating sites. We abstracted data using a standard form including program, ITE raw score, year of ITE administration, US Medical Licensing Exam Step 1 score, Step 2 Clinical Knowledge (CK) score, and gender. We performed univariable and multivariable linear regression to explore differences between intervention and control groups.

**Results:**

We collected data for 180 PGY-1s. Step 1 and Step 2 CK scores were significant predictors of ITE in univariable analyses (both with *P* < 0.001). After accounting for Step 1 and Step 2 CK scores, we did not find F1 implementation to be a significant predictor of ITE score, *P* = 0.83.

**Conclusion:**

Implementation of F1 curricula did not show significant changes in performance on the ITE after controlling for important variables.

## INTRODUCTION

Residency programs provide education and training to develop competent physicians. Board certification in emergency medicine (EM) requires completion of an Accreditation Council for Graduate Medical Education (ACGME)-accredited training program and a passing score on the Qualifying Examination (QE) and Oral Certification Examination (OCE) administered by the American Board of Emergency Medicine (ABEM).^1^ ^,^ ^2^ The ABEM In-training Examination (ITE) is an important tool used by training programs to assess medical knowledge and prepare residents for the QE.^1^ ^,^ ^3^ The ITE is designed to reflect the content of the Model of Clinical Practice of Emergency Medicine (EM Model) and has predictive value in estimating the likelihood of individual residents passing the QE.^3^ Prior literature suggests that clinical exposure alone leaves significant gaps in fundamental knowledge defined by the EM Model.^4^ Residency didactic curricula provide an opportunity to supplement core knowledge; however, the best methods for providing instruction outside of the clinical setting and preparing trainees for successful performance on the ITE are unknown.

Foundations of Emergency Medicine (FoEM) is a national, free, open-access, online EM curriculum that has been widely adopted in the United States.^5^ ^,^ ^6^ FoEM became available in 2015; registration for use of FoEM courses for the 2022–2023 academic year included 237 registered educational programs, serving 6,326 resident physicians.^5^ ^,^ ^6^ FoEM offers standardized, level-specific, core content for EM residents using learner-centric educational strategies that have been shown to benefit learning such as small-group discussion, peer learning, and individualized guidance.^5^ ^–^ ^11^ Foundations I (F1) is a flipped classroom, case-based course targeting postgraduate year (PGY)-1 residents that includes a 30-unit, systems-based curriculum of fundamental content in the EM Model.^5^ ^,^ ^6^ ^,^ ^12^ Prior literature demonstrates positive effects of the flipped classroom model on learning outcomes.^13^ ^–^ ^15^ The F1 curriculum includes curated self-study resources called “Learning Pathways” for learners to review prior to didactic meetings, in which residents work through multiple F1 cases with a knowledgeable facilitator providing information in an oral-boards style format.^6^ The F1 summarizes essential learning points and shares them with learners to fill knowledge gaps and allow for spaced repetition.^6^ Although the F1 curriculum is not specifically designed for ITE review, third-party paired assessments for each unit have been available for use since 2017.^6^

Limited outcome data of FoEM F1 established quality and demonstrated high satisfaction among faculty leaders and resident learners.^5^ ^,^ ^6^ However, there has not been an assessment of objective measures such as medical knowledge and ITE performance This information can provide a more comprehensive assessment of the value of implementing such a program. In this study, we sought to evaluate the effect of F1 course implementation on ITE performance in the PGY-1 year. We hypothesized that implementation of the structured F1 curriculum would lead to improved performance on the ITE.

## METHODS

We performed a retrospective cohort study of ITE data collected from PGY-1 residents at four ACGME- accredited EM residency programs in the United States before and after implementation of the FoEM F1 curriculum. We selected participating sites that were geographically diverse and included 3- and 4-year training formats. We collected data in December 2021. All PGY-1 residents at participating sites during the study period were eligible to participate. We excluded PGY-1 residents who were missing data.

We determined that to detect a 5% difference in ITE score with 80% power and an alpha of 0.05, we would need to enroll 81 participants in each group (control and intervention) for a total of 162 participants. Our control group consisted of data from PGY-1 residents for the two years prior to implementation at each site. Our intervention group consisted of data from PGY-1 residents for the two years after implementation at each site. Year of F1 implementation ranged from 2015–2018 at participating sites. The lead author from each site abstracted data using a standard form that included program, ITE raw total score, year of ITE administration, United States Medical Licensing Examination (USMLE) Step 1 score, USMLE Step 2 Clinical Knowledge (CK) score, and resident gender. Prior to data abstraction, the author group read each item on the form aloud and trialed abstracting a small portion of representative data to ensure clarity of meaning and consistency in process.

We calculated descriptive statistics for demographic data and ITE performance. We performed regression analyses to explore differences between the intervention and control groups. We first performed univariable linear regression analyses for variables including implementation of F1, residency program, year of ITE administration, USMLE Step 1 score, USMLE Step 2 score, and resident gender with ITE raw score as our outcome of interest. We included variables with a *P*-value < 0.1 in the univariable regression in a multivariable linear regression with the same outcome variable.We considered variables with a *P*-value of < 0.05 in the multivariable model as statistically significant. We performed all analyses in SPSS v 27.0 (IBM Corporation, Armonk, NY).

This study was approved by the Institutional Review Board of the David Geffen School of Medicine at UCLA.

## RESULTS

We abstracted data from a total of 224 interns. We excluded 44 interns who were missing data. We analyzed data from 180 interns (88 pre-implementation and 92 post-implementation) who had complete data. The demographics of participants with complete data are shown in Table 1. The mean ITE raw score for interns in the control group was 72.15 ± 6.72. The mean ITE score for interns in the intervention group was 72.74 ± 7.93. In the univariable regression analyses, only USMLE Step 1 and USMLE Step 2 CK scores yielded *P*-values of < 0.1 (Table 2). Because our hypothesis centered on the impact of implementation of the F1 curriculum on ITE scores, we forced this variable as the last variable after block entry of variables of USMLE Step 1 score and USMLE Step 2 CK score in the multivariable regression analysis, despite it having a *P*-value of 0.59 in the univariable analysis. After controlling for Step 1 score and Step 2 CK score, F1 implementation was not a significant predictor of ITE score, R square change = 0, *P* = 0.83. The data satisfied all assumptions.

**Table 1.** Demographic data of participating interns.

	Control group n (%) total n = 88	Intervention group n (%) total n = 92
Gender		
Male	32	31
Female	56	60
Non-binary	0	1
Mean USMLE Step 1 score (SD)	232 (14.26)	232 (15.59)
Mean USMLE Step 2 score (SD)	244 (17.02)	246 (14.54)

*USMLE*, United States Medical Licensing Examination.

**Table 2.** Results of univariable regression analysis of recorded variables.

Variable	*P-*value
Implementation of Foundations F1 curriculum	0.59
Residency program	0.22
Year of ITE administration	0.14
USMLE Step 1 score	<0.001
USMLE Step 2 CK score	<0.001
Resident gender	0.24

*USMLE*, United States Medical Licensing Examination; *ITE*, in-training exam; *CK*, clinical knowledge.

## DISCUSSION

Our study demonstrates that both Step 1 and Step 2 CK were significant predictors of ITE score. This is consistent with prior literature in multiple specialties demonstrating associations between USMLE scores and ITE performance.^16^ ^–^ ^19^ We found that our intervention group had a slightly higher raw ITE scores however, after controlling for USMLE scores, this increase was not statistically significant, despite being adequately powered. This was somewhat surprising given that F1 provides a consistent structure and comprehensive coverage of content in the EM model and also incorporates teaching methods that have been shown to enhance learning.^2^ ^,^ ^6^ ^–^ ^11^ However, our results align with previous studies, which have demonstrated that changes in curriculum were not associated with significant differences in ITE performance.^20^ ^,^ ^21^ Specifically, converting an hour of synchronous didactic conference to asynchronous learning, and converting conference lectures to small group, “flipped-classroom” style learning have previously been found to have no significant effect on ITE scores.^20^ ^,^ ^21^

It is important to note that the objective of F1 is to improve EM core knowledge and application in the clinical environment and is not specifically targeted towards ITE test preparation or performance. Additionally, performance on the ITE may not comprehensively represent learner knowledge of EM. This may be one reason that we did not find significant changes in ITE performance. Additionally, variable implementation and usage of F1 at differing programs could influence potential gains. Although the FoEM courses are standardized, participating programs must address their own unique needs and barriers; this may result in variability in course implementation, including variable use of flipped-classroom style asynchronous resources and paired assessments. It is also important to note that the ITE is administered in February of each year; thus, participating PGY-1 residents in this study were only exposed to approximately seven months of the year-long F1 curriculum prior to the ITE.

It is possible that additional improvements may be seen with additional time spent in the curriculum. The nonsignificant improvement seen in this study may be augmented with implementation of Foundations II (F2), which is designed for PGY-2 residents, and Foundations III (F3), which is designed for PGY-3 and PGY-4 residents. These outcomes merit further investigation. While our study did not find a significant increase in ITE scores compared to standard curricula, it was not worse than standard practice and has additional benefits of a free, standardized, pre-packaged, high-quality, adaptable format with user acceptability.^6^

Overall, the results of this study provide important insights for both the numerous programs already using FoEM and those EM residencies considering incorporating it into their training programs.^6^ In addition to prior feasibility and user acceptability data, this study provides an evaluation of objective outcomes, namely knowledge, the first level in Miller’s pyramid of clinical competence.^6^ ^,^ ^22^ There are still many unanswered questions. Further investigation into the effect of the F1 curriculum on ABEM QE and OCE performance should be pursued. Additionally, as FoEM is designed to support knowledge application in the clinical space, future work could evaluate the impact of FoEM on other domains of resident performance.

## LIMITATIONS

This study has limitations. There may be confounders not accounted for in our analysis that could have influenced results. We did not collect data on specific ITE preparation curricula at participating sites, individual usage of external ITE preparation materials outside of training program curricula, time spent using F1 curriculum, use of paired assessments, total number of F1 units completed by participating residents, or time spent studying for ITE in general. However, to the best of our knowledge, there were no other major changes to the site’s didactic curriculum or methods of preparing trainees for the ITE during the study period. Although the F1 course includes standardized content, participating programs must address their own unique needs and variables that impact the consistency of course administration. There may be differences in the personnel who deliver the content, attendance requirements, etc, which are not accounted for in our study. The results seen in this study may not transfer to other sites where adherence to implementation guidelines is more or less consistent.

## CONCLUSION

Our study suggests that the FoEM F1 curriculum is not associated with significant changes in performance on the ITE in EM training programs after controlling for important variables. These results may inform the use and implementation of FoEM courses in EM training programs.
